# Health-related quality of life in patients with colorectal cancer in the palliative phase: a systematic review and meta-analysis

**DOI:** 10.1186/s12904-021-00837-9

**Published:** 2021-09-16

**Authors:** Ida Røed Flyum, Seila Mahic, Ellen Karine Grov, Pål Joranger

**Affiliations:** 1 Department of Nursing and Health Promotion. Faculty of Health Sciences, OsloMet – Oslo Metropolitan University, P.O. Box 4, St. Olavs plass, NO-0130 Oslo, Norway; 2 Department of Bachelor in Nursing, Lovisenberg Diaconal University College, Lovisenberggata 15B, NO-0456 Oslo, Norway; 3 Centre of Diaconia and Professional Practice, VID Specialized University, P.O. Box 184 Vinderen, NO-0319 Oslo, Norway

**Keywords:** Colorectal neoplasms, Palliative care, Nursing, Health-related quality of life, Systematic review, Meta-analysis

## Abstract

**Background:**

The occurrence of colorectal cancer has doubled over the last 50 years and many people are living with the disease in the palliative phase. Therefore, it is important that healthcare personnel have knowledge about the patient’s health-related quality of life (HRQoL). The aim of this review is to investigate how HRQoL is reported by means of different measures for patients in the palliative phase of colorectal cancer and examine which sociodemographic and clinical factors are associated with the mean scores reported for HRQoL.

**Method:**

A systematic review and meta-analysis using forest plots in STATA were conducted. The databases MEDLINE, CINAHL, Embase, Amed, and SveMed+ were used for the systematic searches with combinations of terms for colorectal cancer, the palliative phase and HRQoL. The Cochrane handbook and the PRISMA checklist from 2009 were utilised.

**Results:**

In total, 710 articles were identified. Eleven quantitative studies met the inclusion criteria and six were included in the meta-analysis. Five of the 11 studies had a longitudinal design, while the other six had a cross-sectional design**.** The meta-analyzes shows that the average HRQoL in palliative phase was 62.9 (56.8–69.0) 15D was 0.76 (0.73–0.79), EQ-5D was 0.67 (0.62–0.73), and VAS was 64.1 (53.7–74.4). Multiple sociodemographic and clinical variables were associated with HRQoL and a higher prevalence of common cancer symptoms were reported than gastrointestinal symptoms.

**Conclusion:**

This systematic review revealed that patients with colorectal cancer report low HRQoL. Furthermore, it shows that what affects HRQoL is complicated, including multiple clinical and sociodemographic variables. This underlines the need for further research. To ensure the best possible care, it is important that all healthcare professionals have easy access to knowledge about HRQoL in patients with colorectal cancer, and what impacts it in the last phase of life.

**Supplementary Information:**

The online version contains supplementary material available at 10.1186/s12904-021-00837-9.

## Background

Cancer is one of the most common diseases in the world, and 17 million new cases of cancer were registered worldwide in 2018 [1]. It is estimated that one in three Norwegians will develop cancer before the age of 75. For those who develop cancer in old age, the colorectal site is one of the top three cancer types in terms of incidence, and Norway is one of the countries with the highest rates of such cancer in the world [2]. In 2019, 4295 new cases of colorectal cancer (CRC) were registered in Norway [3]. The term CRC refers to colon cancer, rectum cancer or both [4]. Despite the fact that the five-year survival rate has increased consistently since 1970, more people are living longer with the disease in the palliative phase, a phase in which they are no longer responding to curative treatment, and life-prolonging approaches, symptom management and optimising quality of life (QoL) take over as goals for treatment and care [3, 5]. According to the World Health Organization, the goal of palliative care is symptom management and promoting QoL for patients and their families [6]. QoL includes the individual’s perception of their personal situation in their own life such as physical, social, mental and spiritual dimensions [7]. HRQoL is one component of QoL [8]; QoL with a specific health component [9]. The term HRQoL refers to a person’s subjective rating of their satisfaction with general health, and their level of well-being [9]. Since HRQoL is a subjective measure, one of the best ways to measure people’s subjective HRQoL is through questionnaires or patient-reported outcomes (PROMS) – the gold standard in assessment. Several different psychometric tested questionnaires exist [9].

The Norwegian strategy for cancer treatment and care also illustrates the importance of maintaining the best possible QoL for cancer patients [10]. To achieve this goal, it is important to relieve the patient’s physical, psychosocial and spiritual symptoms, and to support the families and next-of-kin [6]. According to the International Council of Nurses (ICN), all nurses are responsible for providing care and alleviating suffering [11], which includes contributing to a natural and dignified death. In order to provide care and to maintain HRQoL even in the palliative phase, nurses must have knowledge and understand what is important for the patient in the palliative phase, what creates security and how to approach people in their final phase of life. The most common symptoms experienced by patients in the palliative phase include: pain, fatigue, depression, anxiety, confusion, breathlessness, insomnia, nausea, constipation, diarrhea and loss of appetite [12]. These symptoms affect the patient’s HRQoL, and may be due to their current condition, other chronic diseases and previous treatment such as chemotherapy [13]. Before starting this review, we searched for systematic reviews in the database of the Center for Reviews and Disseminations (CRD) and in the Prospero database for ongoing reviews [14]. We also searched in the McMasterPLUS database where we identified one UpToDate article regarding the HRQoL of patients with CRC. However, the article focused only on patients who no longer had any signs of active disease [15]. Several reviews with a similar focus also exist i.e., HRQoL in patients with CRC. However, these reviews focus on survivors, not on patients in the palliative phase [16–19], or the studies aim to test different interventions [20]. As described, patients in the palliative phase have a lot of unique and distressing symptoms compared to survivors or patients in active treatment. Therefore, the symptoms and HRQoL of patients in the palliative phase of CRC are not necessarily comparable to that of the survivors of CRC. Since cure is not an option for patients in the palliative phase, HRQoL is often considered the outcome for treatment and follow-up, and thereby especially important to review for this patient group. To our knowledge, no systematic reviews have been published in the last 10 years about HRQoL in patients with CRC in the palliative phase. To close this gap, this study will therefore focus on HRQoL in patients in the palliative phase of CRC. The results will give clinicians easier access to relevant and quality-controlled research, thereby giving them a better opportunity to optimise treatment and care for these patients.

The aim of this systematic review is to investigate how HRQoL is reported by means of different measures for patients in the palliative phase of CRC, and examine which sociodemographic and clinical factors are associated with the mean scores reported for HRQoL.

For this review, the following research questions were formulated: (a) What questionnaires are used to measure HRQoL in the different studies? (b) What was the average score for HRQoL for each of the included questionnaires? (c) Which symptoms are reported to impact the patients? (d) Which sociodemographic and clinical factors are found to be statistically significantly correlated with the average HRQoL score in each study?

## Method

Our research team conducted a systematic review and meta-analysis inspired by the Cochrane handbook [21]. The guidelines for systematic reviews and meta-analyses (PRISMA), including the checklist (see Additional file 2) and flowchart were also followed throughout the process [22].

### The systematic search

A systematic search was performed in MEDLINE (Ovid), CINAHL (EBSCOhost), Embase (Ovid), Amed (Ovid), and SveMed+. MeSH terms and keywords for CRC (e.g., colorectal cancer, colon carcinoma, colorectal neoplasms), the palliative phase (e.g., palliative therapy, terminal care, palliative, incurable) and HRQoL (e.g., quality of life, wellbeing, vitality) were included in the search strategy. All the systematic search details are presented in additional file 3. The searches were filtered by publishing year only, 2009–2019. We only included studies from the last 10 years because the field of palliative medicine and care is rapidly changing, and new medicines and methods will have influenced the patients’ HRQoL and the most recent research will therefore be the most relevant for this review. Updated searches were executed for MEDLINE, CINAHL and Embase in March 2020. These three databases were selected for updated searches in communication with the specialist librarian because, together, they cover most of the medical and nursing research and it is therefore likely that any new articles would be identified by this strategy.

### Selection of eligible studies

Two researchers (IRF and SM) separately reviewed all the references by abstract and full text. This is in accordance with the PRISMA standard of systematic reviews [22]. The studies were included if they fulfilled the following criteria: [1] sample over 18 years of age; [2] focused on CRC, the palliative phase and HRQoL; [3] reported results of different patient groups, i.e., different cancer types and disease stages, separately; [4] written in Norwegian, Swedish, Danish or English; [5] original study; and [6] quantitative study design except RCTs. The RCT design was excluded because it has been criticised for being less transferable to real-world settings and may cause artificially positive results by including fewer severely sick individuals [23, 24]. Disagreements on eligibility were resolved by discussion and consulting the full text if necessary. The researchers only disagreed about a few articles, and all of these disagreements were resolved by initial discussion.

### Assessing the quality of the studies

In total, 12 quantitative studies were assessed according to the Critical Appraisal Skills Programme (CASP) for cohort studies [25]. Additional file 4 shows the CASP checklist in more detail. Each study was assessed by three researchers (IRF/SM/EKG and IRF/SM/PJ) to minimise the risk of bias and to increase the validity of the review [23]. The plan was to discuss disagreements and resolve them by consulting with a fourth researcher and reaching consensus through discussion; however, this was not necessary because we agreed on the decisions. The CASP criteria were selected from the appraisal tool and included the factors: [1] a clearly focused issue; [2] an acceptable recruitment strategy; [3] accurate measurement of exposure to minimise bias; [4] accurate measurement of outcome to minimise bias; [5] identifying all important confounding factors and accounting for them in design and analysis; [6] complete follow up of the subjects; [9] is the result believable; [10] relevance for the local population/situation [25]. A score of 8 for the longitudinal studies was considered high, 6–7 moderate and a score of < 6 was considered low. For the cross-sectional studies, a high score was 7, moderate 5–6 and low < 5.

### Data extraction and analysis

In this analysis, we included studies that reported the HRQoL of patients in the palliative phase of CRC. Of the 11 studies included in this review, six studies had appropriate data for the meta-analysis [26–31]. From these six studies, a meta-analysis was conducted, following both the process described in the Cochrane handbook [21] and the reference manual for STATA 16 [32]. For the studies which include multiple measuring times, we have utilised the second line data. For Adamowicz and Baczkowska-Waliszewska [29] and Stein et al. [30], we used the post-treatment and post-progression data.

The study characteristics of each included study and data on which variables were included in the analysis of correlation with the HRQoL score, as well as the statistical significance in each article (Table 4), were extracted and controlled by two researchers (IRF and SM). Data on mean HRQoL, SD, number of participants and type of questionnaire were extracted from each study by two researchers separately (IRF & SM) and controlled by a third researcher (PJ). From this, SE was calculated. The summarised data were rechecked for transcription errors. Data were then managed using Excel version 1902, and all data analysis was carried out in STATA version 16.

The heterogeneity between studies was primarily evaluated by I^2^ values, reference 0–100%, where 25% indicates low heterogeneity, 50% moderate and 75% indicates high heterogeneity [33]. In addition, the value for Cochran’s Q is referred to when appropriate. To explore the heterogeneity, subgroup analyses were performed based on comparison of outcomes for the individual questionnaires. Forest plots were created, showing the effect estimate, level of variability around the estimate for each study and the weight given to each study in the meta-analysis along with the overall result of all studies together [34]. Funnel plots were also created, so that potential publication bias and small-study effects could be assessed visually [32].

Five studies also included the symptom scale from the QLQ-C30 questionnaire but two of these studies only included graphs, not the numerical data [27, 28]. The authors of these articles were contacted but did not respond and some relevant data were therefore not available. Data from the three studies with numerical data were collected (by IRF and SM) and a forest plot was created (Fig. 5). *P-*values < .05 were considered statistically significant [23].

## Results

**Fig. 1:**
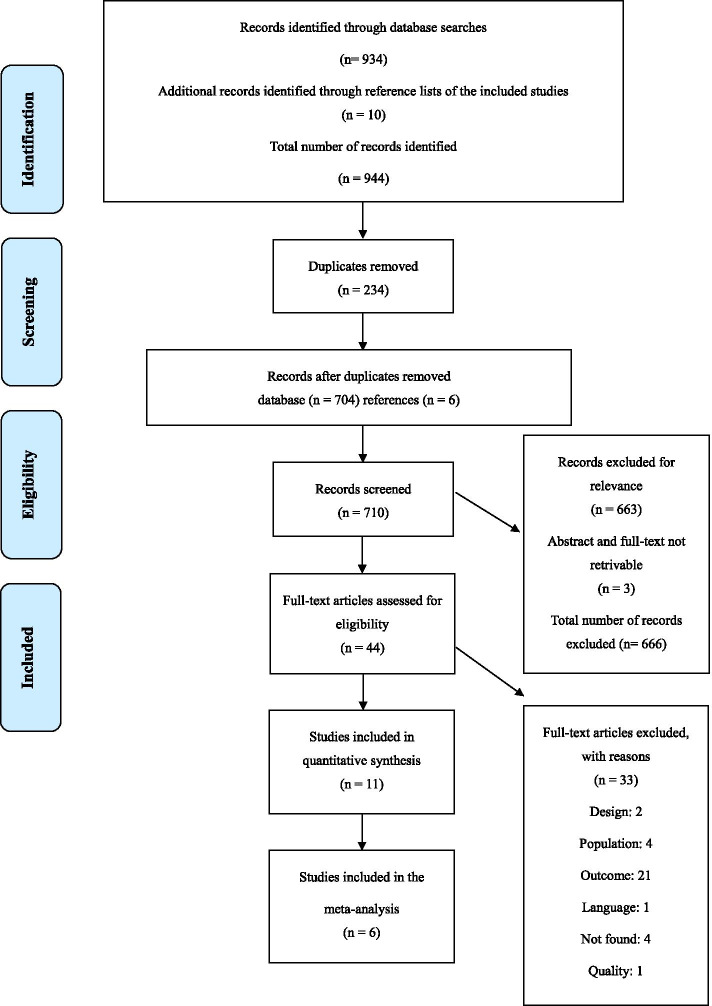
Flowchart of the selection process

In total, we identified 710 potentially relevant articles, after excluding duplicates. Out of these, 11 were deemed eligible. No relevant articles were identified in the updated searches executed in March 2020. The selection process is shown in Fig. 1. Through the analysis for risk of bias, no publication bias was identified. Two researchers (IRF and SM) collected the characteristics of each included article, shown in Table 2. The cumulative sample of patients with CRC in all disease stages was 4629. This review focuses on patients in the palliative phase and therefore the relevant sample is 839. Five of the studies had a longitudinal design, while the other six had a cross-sectional design. The studies also differ in their samples with respect to the level of functioning and the number of deaths or other dropouts during data collection. Moreover, an overview was made of the mean HRQoL scores in different disease stages for all studies included in the meta-analysis, which is presented in Table 5, see additional file 1 for details.

### Results of the quality assessment

Our review only include studies with moderate or high quality (Table 1). Only one study: The estimation of quality of life of the hospitalized terminally ill palliative patients with lung, breast, colon or prostate cancer, by Jasinska et al. [35] was excluded because of methodological weaknesses. This study had an average CASP score (6/8); however, had only included two individuals in the colorectal group. Eleven studies were included [26–31, 36–40], and these are presented in Table 2.

**Table 1:** Quality assessment of included studies

Reference	Criteria								Tot	Quality
	1	2	3	4	5	6	9	10		
Adamowicz [29]	+	+	+	+	+	+	+	+	8/8	High
Asplund [36]	+	+	+	+	+	/	+	+	7/7	High
Färkkilä [28]	+	+	+	+	+	/	+	+	7/7	High
Färkkilä [27]	+	+	+	+	+	/	+	+	7/7	High
Jasinska [37]	+	+	+	+	–	+	+	+	7/8	Moderate
Jasinska [35]	+	+	+	+	–	–	+	+	6/8	Moderate
Kim [38]	+	+	+	+	+	+	+	+	8/8	High
Koskinen [39]	+	+	+	+	+	/	+	+	7/7	High
Mayrbäurl [31]	+	+	+	+	–	+	+	+	7/8	Moderate
Selby [40]	+	+	+	+	–	+	+	+	7/8	Moderate
Stein [30]	+	+	+	+	+	/	+	+	7/7	High
Teker [26]	+	+	+	+	–	/	+	+	6/7	Moderate

Question 6 is not relevant for the cross-sectional designs, and this is symbolised by (/). Questions 11 and 12 in the CASP tool are not relevant for the quality score and are therefore omitted from the table

High quality for the cross-sectional studies = 7, moderate = 5–6, low < 5. High quality for the longitudinal studies = 8, moderate = 6–7, low < 6. Jasinska et al. [35] is included in the table, however, excluded for further analyses because of methodological weakness (see ‘Results of the quality assessment’ for details)

**Table 2:** Presentation of included studies

Reference	Aim	Design	Population/time	ECOG/time before death/stage	Questionnaire
Adamowicz (2018) [29] Poland Journal of Cancer Education	To investigate which environmental factors, incl. Unconventional methods of treatment and dietary supplementation, influenced patients’ QoL.	Prospective	*N* = 330 palliative colon cancer patients Data collection Jan. 2010 to Dec. 2016	50% had an ECOG performance status of 1–2.	QLQ-C30
Asplund (2017) [36] Denmark &Sweden International Journal of Colorectal Disease	To investigate the association of intrusive thoughts and the patients’ sense of coherence with pretreatment QoL in patients with newly diagnosed rectal cancer.	Cross-sectional	*N* = 1085 patients with rectal cancer, all stages. *N* = 73 palliative patients Data collection Feb. 2012 to Sep. 2015		VAS
Färkkilä (2013) [28] Helsinki, Finland. Colorectal Disease	To assess the HRQoL among various disease states of CRC in real-world settings using three standard instruments and to compare it with the HRQoL of the general population. Furthermore, to explore clinical and demographic factors determening HRQoL in CRC.	Cross -sectional	*N* = 508 CRC patients *N* = 41 CRC palliative patients Data collection Oct. 2009 to Feb. 2011.		EQ-5D VAS QLQ-C30 15D
Färkkilä (2014) [27] Helsinki, Finland Quality of Life Research	To explore HRQoL and assess utility in BC, PCa and CRC patients during the final stages of their disease, to compare the results obtained by different HRQoL instruments, and to explore factors related to impaired HRQoL.	Cross-sectional	*N* = 114 *N* = 57 CRC Data collection Sep. 2009 to Apr. 2011.	Months until death for the CRC group: < 3 = 17 (30%) 3–6 = 15 (26%) > 6 = 25 (44%)	EQ-5D VAS QLQ-C30 15D
Selby (2010) [40] Canada Palliative Medicine	To track changes in symptoms and RSCL subscale scores from presentation to 3 months of follow-up.	Prospective, Longitudinal	*N* = 35 patients with non-curable metastatic cancer *N* = 19 mCRC Data collection Mar. 2006 to Mar. 2008	ECOG of all included patients: 0 = 14.3% 1 = 28.6% 2 = 25.7% 3 = 28.6% 4 = 2.9% During the study, 25 patients died.	Edmonton Symptom Assessment System (ESAS) Rotterdam Symptom Checklist (RSCL)
Stein (2014) [30] The Netherlands and the United Kingdom International Journal of Colorectal Disease	To elicit EQ-5D utility values from real-world mCRC patients receiving second and subsequent lines of therapy both pre- and post-progression in a real-world setting.	Cross-sectional	*N* = 75 mCRC N = 33 post- progression/ palliative Data collection Apr. 2012- Dec. 2012	ECOG of all included patients (for palliative): 0 = 24% (21.2%) 1 = 66.7% (66.7%) 2 = 9.3% (12.1%)	EQ-5D VAS
Teker (2015) [26] Turkey JBUON- Open Acess Journal amied at the diffusion of scientific knowledge in oncology.	To evaluate the QoL in CRC patients undergoing chemotherapy and to explore the relationship between QoL and patient characteristics and to evaluate the relationship between QoL and different chemotherapy regimens.	Cross-sectional	*N* = 101 CRC N = 73 CRC palliative Timeframe unknown	ECOG: All patients between 0 and 2	QLQ-C30
Mayrbäurl (2016) [31] Austria Support Care Cancer	To compare HRQoL as reported by patients measured by computer-assisted completion of validated questionnaires in patients with nonresectable advanced CRC while they were undergoing treatment with several palliative chemotherapy lines.	Prospective, longitudinal	*N* = 100 Patients with nonresectable advanced CRC. Data collection Feb. 2007- Sep. 2011	25% of the patients died during the first year of the study. 29% died during the second year and 26% died during the third year.	QLQ-C30
Jasinska (2010) [37] Poland Contemporary Oncology	To assess the effectiveness of palliative care during the hospitalization period in palliative oncological, end-of-life patients with lung, prostate, breast and colon cancer. Considering the linkage between the outcomes and type of tumor.	Prospective, longitudinal	N = 41 *N* = 16 CRC palliative Data collection Feb. 2007 to Apr. 2009	ECOG of all included patients: 1 = 0% 2 = 4.88% 3 = 53.66% 4 = 41.46% 20 patients died during the study. 37.5% of the 16 CRC patients died.	EORTC QLQ-C15-PAL
Kim (2013) [38] South Korea Psycho-Oncology	To investigate the impact of patients’ awareness of their terminal status on their survival and QoL.	Prospective cohort	*N* = 262 *N* = 56 = metastatic, terminal CRC Data collection Mar. 2009 to Aug. 2011	ECOG of all included patients: 0 = 5.7% 1 = 7.3% 2 = 29.8% 3 = 40.8% 4 = 16.4%	VAS
Koskinen (2019) [39] Finland ACTA ONCOLOGICA	To examine the relationship between financial difficulties and HRQoL among breast, prostate and colon cancer patients in different stages of the disease as well as to calculate the total burden to patients caused by cancer. Also, to identify patient characteristics that are associated with financial difficulties and HRQoL and identify factors affecting the cost.	Cross-sectional	*N* = 1.978 N = 508 CRC N = 41 CRC palliative Data collection 2009–2011		EQ-5D VAS QLQ-C30 15D

### Methods for measuring HRQoL

There are different levels of HRQoL, and the levels presented in this article refer to the conceptual model by Spilker from 1996 [9]. Furthermore, a variety of questionnaires are relevant for measuring HRQoL. The European Organization for Research and Treatment of Cancers’ (EORTC) quality of life questionnaire, QLQ-C30, is a disease-specific questionnaire with focus on cancer, with a possible score range of 0–100, with higher scores indicating better HRQoL. The questionnaire includes questions about five function domains and eight questions about the person’s symptoms [41]. Although the questionnaire is disease-specific, the scores we have used in our meta-analysis refer to the person’s overall perception of their health status and QoL. Therefore, we acknowledge that such scores belong to the category of HRQoL. The study by Aronson et al. [41] considered the psychometric properties (reliability and validity) of the QLQ-C30 among the three language-cultural groups: patients from English-speaking countries, Northern Europe and Southern Europe. The results of the study support the questionnaire as a reliable and valid measure of the QoL of cancer patients in different clinical research settings. The EuroQoL EQ-5D and the 15D questionnaire are two validated questionnaires that measure the individual’s HRQoL. The EQ-5D questionnaire includes questions on the person’s mobility, self-care, pain, usual activities and psychological status, with three possible answers for each item [42]. Feng et al. [43] show in their article that the EQ-5D is a valid and reliable generic HRQoL instrument across a broad range of populations, settings and conditions. The 15D questionnaire consists of 15 dimensions: breathing, mental function, speech (communication), vision, mobility, regular activities, hearing, nutrition, elimination, sleep, distress, discomfort and symptoms, as well as sexual activity. Each dimension is divided into five levels, each with an answer option [44]. Sintonen [44] examines in his study the psychometric properties of acceptability, validity, reliability, responsiveness and discriminatory ability of the 15D instrument, and asserts that its properties are superior in several respects to existing generally used profile and single index score instruments. Both of the questionnaires give a single index score, with a value range between 0 and 1 [42, 44].

The Visual Analog Scale (VAS) is a visual scale where a score of 100 indicates the best imaginable health state and 0 the worst [42]. VAS is also considered a strong, clinically useful, valid and reliable instrument, for its measure of symptom intensity [45].

### Meta-analysis

**Fig. 2:**
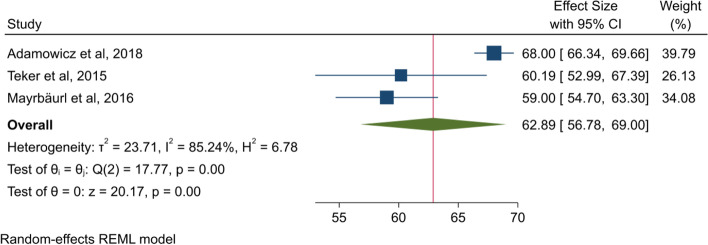
Forest plot for QLQ-C30

We analysed the QLQ-C30 results as one subgroup (Fig. 2). The mean HRQoL score found in this meta-analysis indicates that the patients in the palliative phase of CRC included in the present studies on average scored 2.19 points higher than the reported reference value for patients with recurrent or metastatic CRC using the QLQ-C30 by Scott et al. [46]. The heterogeneity between the studies included in this analysis is high (I^2^ = 85.24%).

**Fig. 3:**
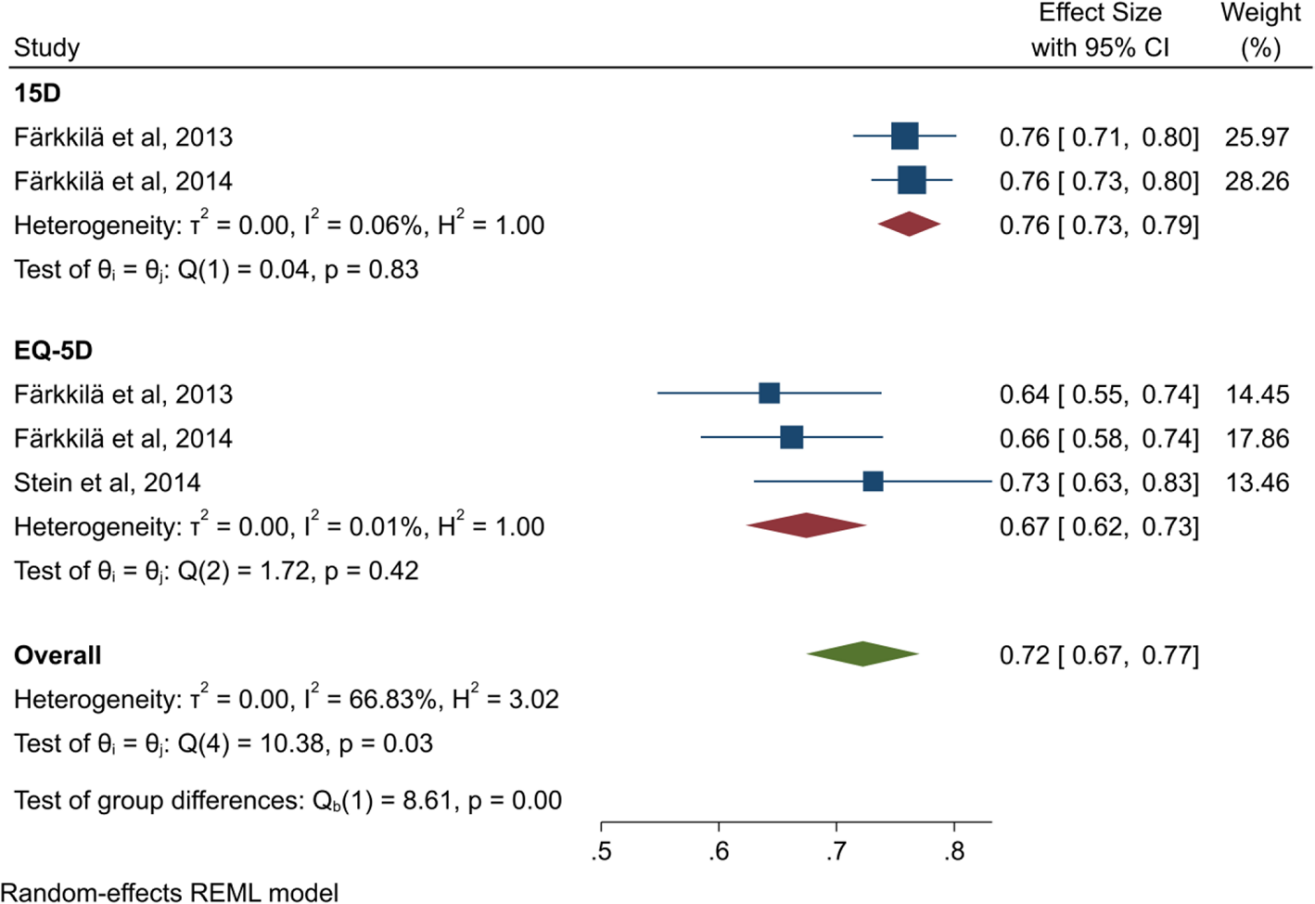
Forest plot for 15D and EQ-5D

As shown in Fig. 3, the heterogeneity is in the moderate to high range for the overall estimate of EQ-5D and 15D (I^2^ = 66.83%). When the questionnaires are analysed separately, the heterogeneity decreases to a low level (I^2^: 15D = 0.06 and EQ-5D = 0.01). This decrease in heterogeneity when the analysis is split into subgroups strengthens the validity of the result from each subgroup. The analysis for the EQ-5D questionnaire shows that the score is 0.083 points lower than the reference score for the general population (+ 75 years) in Denmark [42].

**Fig. 4:**
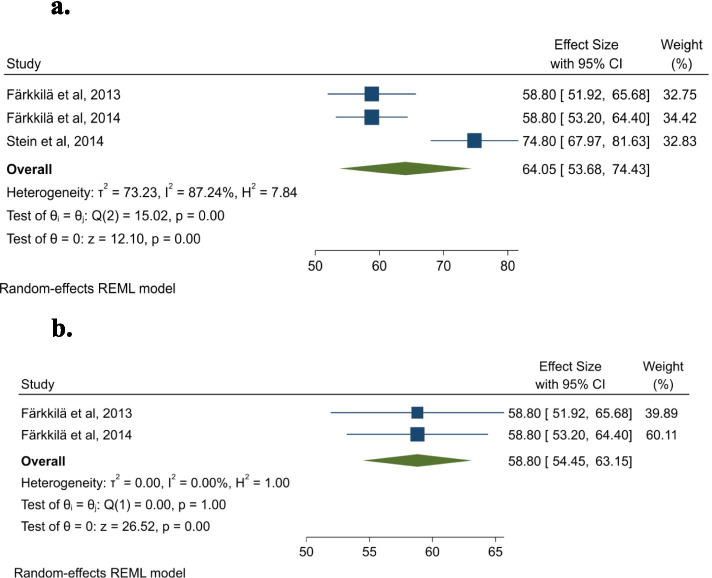
**a** Forest plot for Visual analog scale with Stein et al., [30] included. **b** Forest plot for Visual analog scale (Stein et al., [30] excluded)

Figure 4a shows that our results for VAS were 12.15 points lower than the reference value from Denmark for the general population, 75+ years group [42]. This indicates that this patient group has lower HRQoL compared to the general population. The heterogeneity for this analysis is in the high range (I^2^ = 87.24%). The *p-*value for the Cochran’s Q (*p* < .005) also indicates heterogeneity [33].

### Meta-analysis of the QLQ-C30 symptoms scale

**Fig. 5:**
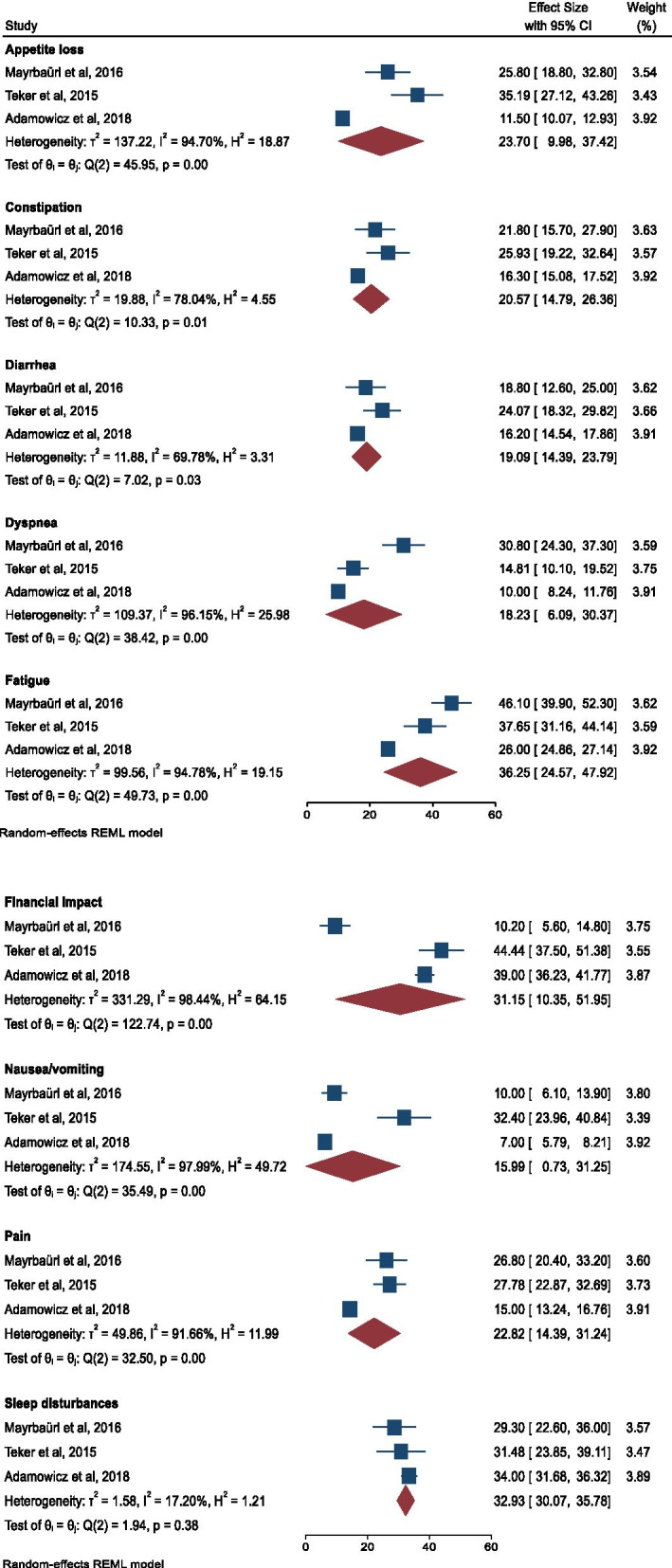
Forest plot for the QLQ-C30 symptom scale

Three of the studies included in this review included data on the symptom scale from the QLQ-C30 questionnaire. The results of a meta-analysis of these data are shown in Fig. 5. Fatigue, financial impact and sleep disturbance are the symptoms that score the highest, with an overall score of over 30 points. Nausea and vomiting are the symptoms with the lowest score, with diarrhea and constipation as third and fourth lowest. Appetite loss and pain are reported with a higher score than the other gastrointestinal (GI) symptoms. Financial impact, nausea and vomiting, pain, appetite loss, dyspnea, constipation and fatigue all have high heterogeneity, over 75%. Diarrhea has a I^2^ value indicating a moderate to high amount of heterogeneity (I^2^ = 69.78%), while sleep disturbance is the only symptom showing low heterogeneity (I^2^ = 17.20%).

### Sensitivity analysis

Our meta-analysis found problems with clinical and methodological heterogeneity related to the article by Adamowicz and Baczkowska-Waliszewska [29]. Methodological because it is not clear which data should be used from the study. While Adamowicz and Baczkowska-Waliszewska [29] report pre- and post-treatment, the other study included in the analysis report on overall HRQoL and symptoms in 1st, 2nd and 3rd line (we use the data from 2nd line). In the sensitivity analysis, we therefore report both for pre- and post-data from Adamowicz and Baczkowska-Waliszewska [29].

There might also be clinical heterogeneity because the patients in the Adamowicz and Baczkowska-Waliszewska [29] article have a longer survival time than normal compared to stage IV patients, and therefore appear to tolerate CRC and/or treatment better. In the sensitivity analysis, we therefore also estimate the result without data from Adamowicz and Baczkowska-Waliszewska [29]. The results of these sensitivity analyses based on QLQ-C30 are shown in Table 3. It is clear that the heterogeneity of the mean HRQoL and many of the symptoms change depending on how data from Adamowicz and Baczkowska-Waliszewska [29] are handled, while the estimates of the mean of e.g., HRQoL vary more modestly, from 62 to 59. If we exclude the data from Adamowicz and Baczkowska-Waliszewska [29], the analysis generally results in a worse mean HRQoL and more negative symptoms. For more details, see additional file 5.

**Table 3:** Sensitivity for meta-analysis based on QLQ-C30

	Including Adamowicz et.al.:data pre-treatment	Including Adamowicz et.al.: data post-treatment	Excluding data from Adamowicz et.al.
Overall HRQoL			
Mean (CI)	62.01 (57.72–66.29)	62.89 (56.78–69.00)	59.31 (55.62–63.00)
Heterogeneity: Q	7.47, *p* = 0.02	17.77, *p* = 0.00	0.08, *p* = 0.78
Heterogeneity: I^2^	69.60%	85.24%	0.00%
Symptoms			
*Appetite loss*			
Mean (CI)	23.61 (9.72–37.51)	23.70 (9.98–37.42)	30.27 (21.08–39.46)
Heterogeneity: Q	45.47, *p* = 0.00	45.95, *p* = 0.00	2.97, *p* = 0.08
Heterogeneity: I^2^	94.67%	94.70%	66.29%
*Constipation*			
Mean (CI)	20.77 (15.49–26.05)	20.57 (14.79–26.36)	23.67 (19.15–28.18)
Heterogeneity: Q	8.43, *p* = 0.01	10.33, *p* = 0.01	0.80, *p* = 0.37
Heterogeneity: I^2^	73.89%	78.04%	0.00%
*Diarrhea*			
Mean (CI)	16.20 (5.86–26.54)	19.09 (14.39–23-79)	21.57 (16.41–26.73)
Heterogeneity: Q	47.50, p 0 0.00	7.02, *p* = 0.03	1.49, *p* = 0.22
Heterogeneity: I^2^	94.23%	69.78%	33.00%
*Dyspnea*			
Mean (CI)	17.97 (5.45–30.49)	18.23 (6.09–30.37)	22.64 (6.97–38.31)
Heterogeneity: Q	41.63, *p* = 0.00	38.42, *p* = 0.00	15.25, *p* = 0.00
Heterogeneity: I^2^	96.33%	96.15%	93.44%
*Fatigue*			
Mean (CI)	34.64 (19.96–49.33)	36.25 (24.57–47.92)	41.93 (33.65–50.21)
Heterogeneity: Q	80.88, *p* = 0.00	49.73, *p* = 0.00	3.40, *p* = 0.07
Heterogeneity: I^2^	96.65%	94.78%	70.62%
*Financial impact*			
Mean (CI)	30.81 (10.25–51.37)	31.15 (10.35–51.95)	27.22 (−6.34–60.77)
Heterogeneity: Q	115.79, *p* = 0.00	122.74, *p* = 0.00	64.98, *p* = 0.00
Heterogeneity: I^2^	98.39%	98.44%	98.46%
*Nausea/vomiting*			
Mean (CI)	15.51 (−0.39–31.41)	15.99 (0.73–31.25)	20.87 (−1.07–42.82)
Heterogeneity: Q	41.26, *p* = 0.00	35.49, *p* = 0.00	22.28, *p* = 0.00
Heterogeneity: I^2^	98.10%	97.99%	95.51%
*Pain*			
Mean (CI)	24.59 (19.89–29.29)	22.82 (14.39–31.24)	27.42 (23.52–31.31)
Heterogeneity: Q	8.15, *p* = 0.02	32.50, *p* = 0.00	0.06, *p* = 0.81
Heterogeneity: I^2^	71.76%	91.66%	0.00%
*Sleep disturbance*			
Mean (CI)	33.27 (28.77–37.76)	32.93 (30.07–35.78)	30.25 (25.21–35.28)
Heterogeneity: Q	4.28, *p* = 0.12	1.94, *p* = 0.38	0.18, *p* = 0.67
Heterogeneity: I^2^	53.09%	17.20%	0.00%

Q: Cochran’s Q. I^2^ = 100% x (Q-dt)/Q, where df is the degrees of freedom and Q the Cochran’s heterogeneity statistics

The forest plot of VAS showed high heterogeneity. One possible explanation for this can be difference in patients’ performance status. Stein et al. [30] enrolled patients from second or subsequent lines of palliative therapy which had an ECOG performance status score of 0, 1 or 2 when initiation of second-line therapy. While Färkkilä et al. [27] only included patients that had died from cancer within 6 months after finishing the questionnaire. From Färkkilä et al. [28], we found no information about the patients’ performance status. Figure 4b shows the VAS score for Stein et al. [30] excluded. The overall VAS score changed from 64.05 to 58.80 and the heterogeneity decreased.

### Sociodemographic and clinical variables influencing HRQoL

Table 4 shows the variables in all the included studies which are statistically significantly correlated with HRQoL. All of the studies that include different cancer types or stages in their sample do not clarify whether the analysis was conducted for the whole group or for the subgroups. We therefore decided to include all the variables but have noted where there are differences.

**Table 4:** Sociodemographic and clinical variables

	Adamowicz (2018) [29]	Asplund (2017) [36]	Färkkilä (2013) [28]	Färkkilä (2014) [27]	Jasinska (2010) [37]	Kim (2013) [38]	Koskinen (2019) [39]	Mayrbäurl (2016) [31]	Selby (2010) [40]	Stein (2014) [30]	Teker (2015) [26]
**Education**	No ^β^	–	–	Yes ^μ (b)^	–	No ^α^	Yes ^μ (b) a^	–	–	–	–
**Age**	No ^β^	–	Yes^d, μ,α (c)^	–	–	No ^α^	Yes ^μ (c)a^	–	No ^Ω^	–	No^a β^
**Sex**	No ^β^	–	–	Yes ^μ,α (b)^	–	–	–	–	Yes ^Ω (c)^	–	–
**Marital status**	–	–	Yes^d, α, (b)^	Yes ^α (b)^	–	–	–	–	–	–	–
**Place of residence**	No ^β^	–	–	–	–	–	–	–	–	–	–
**Financial difficulties**	–	–	Yes^d, μ,α (c)^	Yes ^α (c)^	–	–	Yes ^μ (c)a^	–	–	–	No^a β^
**Stage**	–	Yes ^α (c)^	Yes^d, μ,α (c)^	–	–	–	Yes ^μ (c)a^	–	–	No^d, α^	No^a β^
**Type of Chemotherapy /treatment**	–	–	–	–	–	–	–	–	No ^Ω^	–	No^a β^
**Chemotherapy Line/ Duration of treatment**	–	–	–	–	–	–	–	Yes ^β (c)^	Yes^Ω (c)^ No^∑^	–	No ^β^
**ECOG-score**	Yes ^β (c)^	–	–	–	–	–	–	–	No ^Ω^	–	–
**Targeted treatment**	No ^β^	–	Yes ^α, (c)^	–	No ^π^	–	–	–	No ^Ω^	–	No^a β^
**Smoking status**	No ^β^	–	–	–	–	–	–	–	–	–	–
**Response to treatment**	Yes ^β (b)^	–	–	–	–	–	–	Yes ^β (b)^	–	–	–
**Oncology knowledge**	Yes ^β (b)^	–	–	–	–	–	–	–	–	–	–
**Unconventional treatment**	Yes ^β (c)^	–	–	–	–	–	–	–	–	–	–
**Out-of-pocket cost**	–	–	–	–	–	–	Yes ^μ (c) a^	–	–	–	–
**Total economic costs**	–	–	–	–	–	–	Yes ^μ (c) a^	–	–	–	–
**Comorbidities**	–	–	–	–	–	–	–	–	–	–	No^a β^
**Depression**	–	–	–	Yes ^α (c)^	–	Yes ^α (c)^	–	–	–	–	–
**Feeling of Coherence**	–	Yes ^α (b)^	–	–	–	–	–	–	–	–	–
**Intrusive thoughts**	–	Yes ^α (c)^	–	–	–	–	–	–	–	–	–
**Time from diagnosis**	–	–	–	Yes ^μ (c)^	–	–	–	–	–	–	–
**Hospital stay**	–	–	–	–	No ^π^	–	–	–	–	–	–
**Awareness of terminal status**	–	–	–	–	–	Yes ^α (c)^	–	–	–	–	–
**Metastasis to lungs**	–	–	–	–	–	No ^α^	–	–	–	–	–
**Treatment at enrolment**	–	–	–	–	–	–	–	–	–	No^d^	–
**Treatment at enrolment + stage**	–	–	–	–	–	–	–	–	–	Yes^d (b)^	–

Yes = *p* < .05. No = *p* > .05. --- = Not analyzed

^a^ = Not only patients with CRC in the palliative phase in the analysis

^b^ Indicates a positive correlation between the variable and the HRQoL -score

^c^ Indicates a negative correlation between the variable and the HRQoL -score

^d^ = EQ-5D, μ = 15D, α = VAS, β = QLQ-C30 π = QLQ-C15 Ω = RSCL ∑ = ESAS

Education was analysed for in four studies, but only two showed that higher education was significantly correlated with higher HRQoL using the 15D questionnaire [27, 39]. Age and sex were analysed for in six and three studies respectively. For age, one study using 15D and one study using 15D, EQ-5D and VAS indicated that higher age was significantly correlated with lower HRQoL [28, 39], while sex was significant in two of the studies. One of these studies [27] using VAS and 15D indicates that the female gender was significantly correlated with higher HRQoL. On the other hand, Selby et al. [40] report the female gender to be statistically significantly correlated with a lower score on the psychological subscale of the Rotterdam Symptom Checklist (RSCL).

Five studies analysed for stage and three of them indicate that more severe disease state statistically correlates with HRQoL [28, 36, 39]. Five studies analysed for the effect of targeted treatment, but only one [28] showed a significant negative correlation between HRQoL and radiotherapy. Both studies that analysed for depression found that higher levels of depression were statistically significantly correlated with lower HRQoL [27, 38]. Financial difficulties were associated with lower HRQoL in three [27, 28, 39] of the four studies that analysed for this. Marital status, response to treatment, feeling of coherence, higher levels of oncology knowledge and treatment in correlation with stage were positively correlated with HRQoL.

Adamowicz and Baczkowska-Waliszewska [29] found that a better performance status (ECOG score = 0) indicated a better HRQoL. Selby et al. [40], on the other hand, did not find any correlation between performance status and HRQoL. Mayrbäurl et al. [31] indicate that HRQoL increased from first-line chemotherapy to post first-line, but then decreased from post first-line to post third-line, from 65.3 to 44.2 (*P* < .001). Selby et al. [40] indicate that the ESAS score significantly decreased from the baseline value to the one-month assessment point. The results of the RSCL questionnaire showed no significant change in HRQoL over time. Teker, Demirag, Erdem, Kemal and Yucel [26] found no correlation between the chemotherapy line and HRQoL. The presence of intrusive thoughts, time from diagnosis, awareness of terminal disease and use of unconventional treatment, out-of-pocket cost and total cost were statistically negatively correlated with HRQoL. Comorbidities, staying at a hospital, metastasis to lungs, smoking status, place of residence, type of chemotherapy or treatment and treatment at enrolment did not significantly correlate with HRQoL.

## Discussion

The results of this review show that the average HRQoL score of patients in the palliative phase of CRC are comparable to that of long-term survivors of CRC from previous studies, but lower than for the general population. Multiple sociodemographic and clinical variables were statistically significantly associated with HRQoL. The highest scored symptoms were symptoms generally associated with cancer, such as pain, fatigue and sleep disturbances, while GI symptoms such as diarrhea and constipation were scored somewhat lower. Overall, this review shows that there are both similarities and differences between the HRQoL in CRC survivors and patients in the palliative phase, which is an important finding which healthcare professionals should be aware of when caring for this patient group. Including, but not limited to identification of the patients’ symptoms and tailoring a treatment plan in collaboration with the individual, is essential. The findings from this study are valuable in terms of creating a plan with focus on the most relevant aspects of HRQoL for colorectal cancer patients. The differences found between the two patient groups in this review underpin its importance, given that no review has previously been published on this specific patient group. Furthermore, policy makers need to be aware of these differences to optimize the care strategy offered this patient group through specified guidelines.

### Which HRQoL questionnaires are commonly used and what is the average HRQoL score in the palliative phase of CRC?

With respect to the first research question; which questionnaires are used to measure HRQoL in the included studies, we found that the most common questionnaires used to measure HRQoL in our sample of studies were the QLQ-C30, the 15D, the EQ-5D and the VAS scale. In addition, the ESAS questionnaire, the RSCL and the QLQ-C15-PAL were used in the included studies.

With respect to the question; what is the average score for HRQoL for each of the included questionnaires, we found that compared to the results of Nolte et al. [47], the sample in this review had a lower HRQoL than that of the general population in 13 European countries. On the other hand, the average score for the QLQ-C30 data in this review was only a few points higher than the reported reference value for patients with recurrent or metastatic CRC using the same questionnaire as reported by Scott et al. [46].

Furthermore, heterogeneity is high in the meta-analysis of QLQ-C30. The patients in Adamowicz and Baczkowska-Waliszewska [29] have a longer survival time than normal compared to stage IV patients, and both Adamowicz and Baczkowska-Waliszewska [29] and Teker et al. [26] appear to have healthier samples with an ECOG score between 0 and 2, with over half of the sample in Adamowicz and Baczkowska-Waliszewska [29] scoring 0 (no problems). On the other hand, Mayrbäurl et al. [31] do not refer to an ECOG score, but the median survival time after study inclusion was 21.8 months (51 dropouts due to death). This seems to indicate that this sample was more affected by their illness, which can explain the between-study heterogeneity. In addition to this clinical heterogeneity, we also argue that there is methodological heterogeneity. This is due to certain ambiguities regarding at what stage of the palliative care measurements of QLQ-C30 were performed. The sensitivity analyses we have performed show estimates of what variations in outcome the aforementioned types of heterogeneity can cause.

The large sample size and small confidence interval (CI) identified in the study by Adamowicz and Baczkowska-Waliszewska [29] mean this study is given more weight in the meta-analysis (Fig. 2). Furthermore, this sample has a higher mean HRQoL and thereby raises the average score for the analysis [32]. Both Adamowicz and Baczkowska-Waliszewska [29] and Stein et al. [30] include more female participants compared to the other two studies included in each analysis; Teker et al. [26], Mayrbäurl et al. [31], Färkkilä et al. [27] and Färkkilä et al. [28], respectively. Thus, compared with Nolte et al. [47], our review showed that women report higher HRQoL than men. On the other hand, the review by Bours et al. [16] on survivors of CRC found inconclusive evidence for the association between gender and HRQoL, with several studies associating each gender with lower HRQoL. The performed sensitivity analyses indicate variations in HRQoL score that may have been caused by clinical and methodological heterogeneity. With respect to both QLQ-C30 and HRQoL measurements, we recognise the importance of the information in the original studies about the health state of the patients and when the measurements were performed during the palliative treatment.

We also found that when the 15D and the EQ-5D were analysed together, the heterogeneity was moderate to high, but when analysed separately the heterogeneity decreased to a low level. This indicates that this methodological heterogeneity was caused by the use of different questionnaires [21]. Results for the EQ-5D indicate that our sample had a lower HRQoL (0.083) than the general population (+ 75 years) in Denmark [42]. Furthermore, our results are similar to (0.03 lower than) those of Rodriguez, Hawkins, Berkowitz and Li [48], who investigated the HRQoL in survivors of CRC. This indicates that the HRQoL in palliative CRC patients is similar to that of people no longer struggling with the disease. The comparable HRQoL of these different patient groups can be explained by CRC survivors struggling with late effects as reported by Haggstrom and Cheung [15], as well as depression and anxiety about experiencing recurrence as expressed by the patients in the systematic review by Jansen, Koch, Brenner and Arndt [19].

Similar to the results of EQ-5D, the results of the meta-analysis including Färkkilä et al. [27], Färkkilä et al. [28] and Stein et al. [30] show that the sample measured using VAS had a 12.15-point lower HRQoL than the Danish general population [42]. On the other hand, more than three quarters of the sample in Stein et al. [30] had an ECOG score of 0–1, indicating almost no impact on their performance status. In Färkkilä et al. [27], over half of the sample died within 6 months, indicating that the sample was in a late stage of their disease. This difference in health state can explain why the participants in the Stein et al. [30] study reported a higher average HRQoL.

### Which symptoms are linked to HRQoL in the palliative phase of CRC?

With respect to the analysis linked to the question; which symptoms are reported to impact the patients with CRC in the palliative phase, it is interesting to note that the included patients reported lower GI symptoms, which are often especially associated with CRC, than the more general cancer-related symptoms such as fatigue, pain and sleep disturbance [12]. This can be explained by the fact that the GI symptoms are among the most common symptoms for CRC patients [49], and are therefore well managed. This is somewhat in accordance with the result of the review by Bours et al. [16], which found that GI symptoms was weakly associated with HRQoL in survivors of CRC, while fatigue, anxiety and depression were strongly associated with HRQoL. On the other hand, compared to our results, Bours et al. [16] found that pain was only weakly associated with HRQoL.

Furthermore, the analysis of nausea and vomiting, appetite loss, constipation, dyspnea, fatigue, pain and financial impact were all highly heterogeneous. In all of the heterogeneous analyses, apart from the financial impact variable, Adamowicz and Baczkowska-Waliszewska [29] scored the best out of the three studies. This can be explained by the fact that the sample participants in Adamowicz and Baczkowska-Waliszewska [29] seem to be less affected by their disease. The sample also has a higher percentage of highly educated individuals. Interestingly, similar to the overall HRQoL score discussed above, Adamowicz and Baczkowska-Waliszewska [29] have the highest percentage of women of the three studies included in the symptom analysis but still have the best score despite the female gender often being linked to more symptoms and lower HRQoL as in the study by Nolte et al. [47].

Teker et al. [26] have the highest score on most of the variables as shown in Fig. 5 (excl. Dyspnea, sleep disturbance and fatigue). Overall, this sample has lower education and scores the highest on the financial impact variable, which can explain why these individuals seem to have poorer symptom management. These associations are in accordance with the results of Jansen et al. [19], while Lathan et al. [50] found the association with financial difficulties.

As mentioned, the sample in Mayrbäurl et al. [31] seems to be most affected by their disease, which explains why this sample has the highest dyspnea and fatigue scores. These symptoms are typical of the last part of life as explained in the literature summary by Chang [12]. Mayrbäurl et al. [31] also have the lowest score on financial impact, which can be explained by the fact that the study is from Austria, where there is a lower share of households with impoverishing health spending (health spending that causes the household to drop below the poverty line) as described by the World Health Organization Regional Office for Europe [51], compared to the study by Teker et al. [26] from Turkey and the study by Adamowicz and Baczkowska-Waliszewska [29] from Poland. In accordance with our results, Bours et al. [16] found that lower household income was associated with HRQoL in CRC survivors.

### Which sociodemographic and clinical factors are correlated with HRQoL?

With respect to the question; which sociodemographic and clinical factors are found to be statistically significantly correlated with the average HRQoL score in each study, we found that similar to the results for the average HRQoL, Selby et al. [40] report that the female gender was associated with a lower score on the psychosocial scale of the RSCL instrument. This is in accordance with the results of a study by Paika et al. [52] investigating HRQoL in patients with CRC using the World Health Organization Quality of Life Instrument, Short-Form. Färkkilä et al. [27] on the other hand, found the female gender to be associated with higher HRQoL using VAS and the 15D instrument. The different measuring instruments used and the fact that the psychosocial scale is only a subscale of the full RSCL measurement can explain the difference.

The association between HRQoL and age, more severe disease stage, time since diagnosis, chemotherapy line and response to treatment are substantiated by physiological mechanisms such as age-related homeostenosis [53], more invasive tumours and spreading to other organs or lymph nodes [49] and the side-effects of chemotherapy [15]. The use of targeted treatment (radiation) was associated with lower HRQoL, which is explained by the side effects of radiation [54]. On the other hand, radiation is often considered a palliative treatment to reduce pain and other symptoms [55]. In this study, we did not find any significant association between comorbidities or smoking with HRQoL, which deviates from the results of the review by Bours et al. [16], which found several studies that identified an association between comorbidities and smoking status with lower HRQoL in CRC survivors. Only two studies had analysed for the correlation between the ECOG score and HRQoL in our review. In accordance with the results of Bours et al. [16], who found that poor performance status was associated with lower HRQoL in survivors of CRC, one study in our review found that ECOG score was statistically significant, linking better performance status to higher HRQoL. ECOG is a score that indicates the patient’s performance status, which Chang [12] calls a key indicator of prognosis in patients with terminal disease. This variable should be included more often in studies investigating HRQoL.

The use of unconventional treatment was found to be associated with lower HRQoL. This might of course be explained by the fact that these treatments are not necessarily thoroughly tested and can give unknown side effects. Another possible explanation is that the individual is very ill, and therefore open to any treatment that could help. The impact of the disease might cause the association with lower HRQoL. On the other hand, we could hypothesise that these patients would be hopeful of the unconventional treatment working, and therefore report higher HRQoL.

The association between HRQoL and higher education, being married, higher levels of oncology knowledge and feeling of coherence can be explained by the fact that all of these variables, which Lazarus [56] calls personal resources, affect the individual’s ability to cope with stress in a productive manner. In accordance with our results, Jansen et al. [19] also found that education and higher income were associated with higher QoL. On the other hand, financial difficulties, higher out-of-pocket payments, higher total costs, intrusive thoughts, awareness of terminal disease and more depression can negatively affect the individual’s ability to cope with stress [56], and therefore be associated with lower HRQoL. In accordance with the results in this review, Bours et al. [16] found that depression and feelings of coherence were associated with HRQoL. While Haverfield et al. [57] found financial cost to be a worry for oncology patients.

### Strengths, limitations and future direction

We decided to analyse the symptoms scale for the QLQ-C30 only. The other questionnaires also include information of interest in this area and this is a relevant topic to include in future meta-analyses. We excluded studies with an RCT design, which might have excluded some relevant studies. On the other hand, RCTs tend to include healthier individuals with fewer comorbidities. RCTs are also usually executed in controlled environments with personnel who have specialised knowledge and a heightened focus on observation and care [18]. This can lead to HRQoL being skewed to a higher score than is the case for average CRC patients in palliative care.

The studies included are somewhat heterogeneous with respect to the measurements used, and the sociodemographic and clinical variables of the samples. This affects comparison of the studies. The number of studies included in the review is also limited. However, this is due to a rigorous search with a focus on including the most relevant and recent studies.

The most severely ill patients often decline participation in studies. Non-compliance can be due to many factors, such as ethical considerations or that the patient is too frail to complete the questionnaires. This may have affected the average HRQoL score by excluding patients with the lowest scores. The quality of the studies included was also thoroughly assessed by three independent researchers. The fact that we performed a meta-analysis strengthens the results of this review.

Considering the limitations, and the small number of relevant studies, further research is needed. A meta-analysis should be performed highlighting data from all the instruments including symptoms. ECOG is one variable that should be included more often in studies investigating HRQoL. Since the results for both CRC patients in the palliative phase and CRC survivors show an association between psychological distress and HRQoL, it would also be interesting to understand and elucidate the cause of both groups’ depression or anxiety, linked to their different disease stages. This would give healthcare personnel more knowledge to properly tailor their psychological support to each patient group.

## Conclusion

In this systematic review, we found that QLQ-C30, the 15D, the EQ-5D and VAS were the most commonly used questionnaires for measuring HRQoL in the included studies. Furthermore, we found that the average HRQoL score of patients with CRC in the palliative phase is comparable to that of long-term survivors of CRC from previous studies, but lower than for the general population. The highest scored symptoms were symptoms generally associated with cancer, while GI symptoms such as diarrhea and constipation were scored somewhat lower. Education, financial difficulties and performance status were correlated with the patients’ HRQoL. Unlike previous research, no statistically significant association between sex and HRQoL was found in the studies we analysed.

## Supplementary Information

**Additional file 1: Table 5**. Mean HRQoL-scores in different disease stages. Mean (SD/CI).

**Additional file 2.**

**Additional file 3.**

**Additional file 4.**

**Additional file 5.**

## Data Availability

The data used in this review are collected from the included research articles. A list is shown below: Asplund D, Bisgaard T, Bock D, Burcharth J, Gonzalez E, Haglind E, et al. Pretreatment quality of life in patients with rectal cancer is associated with intrusive thoughts and sense of coherence. International Journal of Colorectal Disease. 2017;32:1639–47 [36]. Adamowicz K, Baczkowska-Waliszewska Z. Prognostic Value of Knowledge of Cancer and Used Unconventional Therapy Methods on Quality of Life in Advanced, Metastatic Colorectal Cancer in Clinical Practice. J Cancer Educ. 2018;06:06 [29]. Färkkilä N, Sintonen H, Saarto T, Jarvinen H, Hanninen J, Taari K, et al. Health-related quality of life in colorectal cancer. Colorectal Disease. 2013;15(5):215–22 [28]. Färkkilä N, Torvinen S, Roine RP, Sintonen H, Hanninen J, Taari K, et al. Health-related quality of life among breast, prostate, and colorectal cancer patients with end-stage disease. Qual Life Res. 2014;23:1387–94 [27]. Jasinska M, Tracz M, Kurczewska U, Orszulak-Michalak D. Assessment of change of quality of life in hospitalized terminally ill cancer patients. Wspolczesna Onkologia. 2010;14(5):333–9 [37]. Kim SY, Kim JM, Kim SW, Shin IS, Bae KY, Shim HJ, et al. Does awareness of terminal status influence survival and quality of life in terminally ill cancer patients? Psycho-Oncology. 2013;22(10):2206–13 [38]. Koskinen J-P, Färkkilä N, Sintonen H, Saarto T, Taari K, Roine RP. The association of financial difficulties and out-ofpocket payments with health-related quality of life among breast, prostate and colorectal cancer patients. Acta Oncol. 2019;58(7):1062–8 [39]. Mayrbäurl B, Giesinger J, Burgstaller S, Piringer G, Holzner B, Thaler J, et al. Quality of life across chemotherapy lines in patients with advanced colorectal cancer: a prospective single-center observational study. Supportive Care in Cancer. 2016;24(2):667–74 [31]. Selby D, Wright F, Stilos K, Daines P, Moravan V, Gill A, et al. Room for improvement? A quality-of-life assessment in patients with malignant bowel obstruction. Palliative Medicine. 2010;24(1):38–45 [40]. Stein D, Joulain F, Naoshy S, Iqbal U, Muszbek N, Payne KA, et al. Assessing health-state utility values in patients with metastatic colorectal cancer: a utility study in the United Kingdom and the Netherlands. International Journal of Colorectal Disease. 2014;29:1203–10 [30]. Teker F, Demirag G, Erdem D, Kemal Y, Yucel I. Quality of life in colorectal cancer patients during chemotherapy in the era of monoclonal antibody therapies. Journal of BUON. 2015;20(2):443–51 [26].

## Abbreviations

HRQoL: Health-Related Quality of Life
QOL: Quality of Life
CRC: Colorectal Cancer
PROMS: Patient Reported Outcomes
ICN: International Council of Nurses
CRD: Center for Reviews and Disseminations
CASP: Critical Appraisal Skills Programme
EORTC: The European Organization for Research and Treatment of Cancers
VAS: The Visual Analog Scale
GI: Gastrointestinal
RSCL: Rotterdam Symptom Checklist
CI: Confidence interval.

## Declarations

Ethics approval and consent to participate:
