# Supplementary material for: Health-related quality of life in patients with colorectal cancer in the palliative phase: a systematic review and meta-analysis
Source: BMC Palliat Care. 2021 Sep 16;20:144. doi: 10.1186/s12904-021-00837-9 (PMC8447559; doi:10.1186/s12904-021-00837-9)
Supplement: Supplementary file 1 — Additional file 1: Table 5. Mean HRQoL-scores in different disease stages. Mean (SD/CI). [file 12904_2021_837_MOESM1_ESM.docx]

Additional file 1: Table 5. Mean HRQoL-scores in different disease stages. Mean (SD/CI)

| Table 5. Mean HRQoL-scores in different disease stages. Mean (SD/CI) | | | | | |  |
| --- | --- | --- | --- | --- | --- | --- |
| Mean HRQoL-score | **Reference** | **Primary treatment** | **Rehabilitation** | **Remission** | **Metastatic** | **Palliative** |
| QLQ-C30 | Adamowicz (2018) | -- | -- | -- | -- | 65 (16.5)/ 68 (15.4) |
|  | Teker (2015) | -- | -- | -- | 58.33 (20.54) | 52.89 (27.98)  60.19 (31.38)  72.22 (23.37) |
|  | Mayrbäurl (2016) | -- | -- | -- | -- | 61.6 (57.7-65.4)  59.0 (54.7-63.3)  50.0 (45.5-54.4)** |
|  | Koskinen (2019) | -- | -- | -- | -- | -- |
| 15D | Färkkilä (2013) | 0.889 (0.090) | 0.877 (0.103) | 0.886 (0.106) | 0.860 (0.090) | 0.758 (0.143) |
|  | Färkkilä (2014) | -- | -- | -- | -- | 0.764 (0.133) |
|  | Koskinen (2019) | -- | -- | -- | -- | -- |
| **Statistical significans is noted by * < .05 ** < .01** | | | | | | |

| Continuing table 5. Mean HRQoL-scores in different disease stages. Mean (SD/CI) | | | | | |  |
| --- | --- | --- | --- | --- | --- | --- |
| Mean HRQoL-score | **Reference** | **Primary treatment** | **Rehabilitation** | **Remission** | **Metastatic** | **Palliative** |
| VAS | Stein (2014) | -- | -- | -- | 74.5 (14.2) | 74.8 (19.7) |
|  | Färkkilä (2013) | 68.1 (22.2) | 77.0 (18.7) | 78.9 (18.0) | 73.9 (17.7) | 58.8 (22.2) |
|  | Färkkilä (2014) | -- | -- | -- | -- | 58.8 (21.4) |
|  | Koskinen (2019) | -- | -- | -- | -- | -- |
|  | Kim (2013) | -- | -- | -- | -- | 50.0** |
|  | Asplund (2017) | -- | -- | -- | -- | 53.0** |
| EQ-5D | Stein (2014) | -- | -- | -- | 0.741 (0.230) | 0.731 (0.292) |
|  | Färkkilä (2013) | 0.760 (0.233) | 0.835 (0.207) | 0.850 (0.207) | 0.820 (0.198) | 0.643 (0.311) |
|  | Färkkilä (2014) | -- | -- | -- | -- | 0.662 (0.298) |
|  | Koskinen (2019) | -- | -- | -- | -- | -- |
| **Statistical significans is noted by * < .05 ** < .01** | | | | | | |
