# Supplementary material for: Health-related quality of life in patients with colorectal cancer in the palliative phase: a systematic review and meta-analysis
Source: BMC Palliat Care. 2021 Sep 16;20:144. doi: 10.1186/s12904-021-00837-9 (PMC8447559; doi:10.1186/s12904-021-00837-9)
Supplement: Supplementary file 3 — Additional file 3. [file 12904_2021_837_MOESM3_ESM.docx]

Additional file 3: Search strategies

Embase (Ovid)

| 1. colon carcinoma/ or colon cancer/ or large intestine carcinoma/ or colon adenocarcinoma/ |
| --- |
| 2. colorectal cancer/ or rectum cancer/ or colorectal carcinoma/ or metastatic colorectal cancer/ |
| 3. hereditary colorectal cancer/ or hereditary nonpolyposis colorectal cancer/ |
| 4. sigmoid cancer/ or sigmoid carcinoma/ |
| 5. colorectal tumor/ or colon tumor/ or rectum tumor/ |
| 6. metastatic colon cancer/ |
| 7. (neoplasm* adj2 Colo*).ti. |
| 8. 1 or 2 or 3 or 4 or 5 or 6 or 7 |
| 9. palliative therapy/ or cancer palliative therapy/ |
| 10. terminal care/ or advance care planning/ or hospice care/ or terminal disease/ |
| 11. terminally ill patient/ or hospice patient/ |
| 12. Palliati*.ti,ab. |
| 13. Incurable.ti,ab. |
| 14. "End of life".ti,ab. |
| 15. "Late?stage".ti,ab. |
| 16. "End?stage".ti,ab. |
| 17. 9 or 10 or 11 or 12 or 13 or 14 or 15 or 16 |
| 18. 8 and 17 |
| 19. "quality of life"/ or "quality of life index"/ or short form 36/ |
| 20. "Quality of life".ti,ab. |
| 21. wellbeing/ or physical well-being/ or psychological well-being/ |
| 22. Well?being.mp. |
| 23. Hope.ti,ab. |
| 24. Vigor.mp. |
| 25. Vitality.mp. |
| 26. 19 or 20 or 21 or 22 or 23 or 24 or 25 |
| 27. 18 and 26 |
| 28. limit 27 to yr="2009 -Current" |
| 29. limit 28 to conference abstracts |
| 30. 28 not 29 |

MEDLINE (Ovid)

| 1. colorectal neoplasms/ or colonic neoplasms/ or sigmoid neoplasms/ or colorectal neoplasms, hereditary nonpolyposis/ or rectal neoplasms/ or anus neoplasms/ or duodenal neoplasms/ or ileal neoplasms/ or jejunal neoplasms/ |
| --- |
| 2. (Cancer adj2 Colo*).ti. |
| 3. (carcinoma adj2 Colo*).ti. |
| 4. (adenocarcinoma adj2 Colo*).ti. |
| 5. (tumor adj2 Colo*).ti. |
| 6. (metastatic adj2 Colo*).ti,ab. |
| 7. 1 or 2 or 3 or 4 or 5 or 6 |
| 8. palliative care/ or terminal care/ or hospice care/ |
| 9. Palliati*.ti,ab. |
| 10. Terminal*.ti,ab. |
| 11. Incurable.ti,ab. |
| 12. "End of life".ti,ab. |
| 13. "Late stage".ti,ab. |
| 14. "End stage".ti,ab. |
| 15. 8 or 9 or 10 or 11 or 12 or 13 or 14 |
| 16. 7 and 15 |
| 17. "Quality of Life"/ |
| 18. Well?being.mp. |
| 19. Hope.ti,ab. |
| 20. Vigor.mp. |
| 21. Vitality.mp. |
| 22. "Quality of life".ti,ab. |
| 23. 17 or 18 or 19 or 20 or 21 or 22 |
| 24. 16 and 23 |
| 25. limit 24 to yr="2009 - Current" |

Amed (Ovid)

| 1. colonic neoplasms/ or colorectal neoplasms/ or rectal neoplasms/ |
| --- |
| 2. (Cancer adj2 Colo*).ti. |
| 3. (carcinoma adj2 Colo*).ti. |
| 4. (adenocarcinoma adj2 Colo*).ti. |
| 5. (tumor adj2 Colo*).ti. |
| 6. (metastatic adj2 Colo*).ti,ab. |
| 7. 1 or 2 or 3 or 4 or 5 or 6 |
| 8. palliative care/ or terminal care/ or hospice care/ |
| 9. Palliati*.ti,ab. |
| 10. Terminal*.ti,ab. |
| 11. Incurable.ti,ab. |
| 12. "End of life".ti,ab. |
| 13. "End stage".ti,ab. |
| 14. "Late stage".ti,ab. |
| 15. 8 or 9 or 10 or 11 or 12 or 13 or 14 |
| 16. 7 and 15 |
| 17. "quality of life"/ |
| 18. Well?being.mp. |
| 19. Hope.ti,ab. |
| 20. Vigor.mp. |
| 21. Vitality.mp. |
| 22. "Quality of life".ti,ab. |
| 23. 17 or 18 or 19 or 20 or 21 or 22 |
| 24. 16 and 23 |
| 25. limit 24 to yr="2009 - Current" |

CINAHL (EBSCOhost)

1. (MH "Colonic Neoplasms") OR (MH "Colorectal Neoplasms") OR (MH "Rectal Neoplasms") OR (MH "Duodenal Neoplasms") OR (MH "Ileal Neoplasms") OR (MH "Jejunal Neoplasms") OR (MH "Sigmoid Neoplasms") OR (MH "Colorectal Neoplasms, Hereditary Nonpolyposis") OR (MH "Anus Neoplasms")

2. TI ((colo*) AND (cancer* or carcinom* or adenocarcinom* or tumor* or metastat*))

3. AB ((colo*) AND (cancer* or carcinom* or adenocarcinom* or tumor* or metastat*))

4. S1 OR S2 OR S3

5. TI (palliati* or terminal* or incurable* or "end of life" or "end stage" or "late stage*")

6. AB (palliati* or terminal* or incurable* or "end of life" or "end stage" or "late stage*")

7. S5 OR S6

8. S4 AND S7

9. (MH "Hospice and Palliative Nursing") OR (MH "Palliative Care") OR (MH "Terminal Care")

10. S1 AND S9

11. S8 OR S10

12. (MH "Quality of Life") OR "quality of life" OR (MH "Psychological Well-Being")

13. ""vigor""

14. (MH "Hope") OR (MH "Optimism") OR "hope*"

15. ""vitality""

16. S12 OR S13 OR S14 OR S15

17. S11 AND S16

18. S11 AND S16 Limit: Published Date: 20090101-20201231 (The search was executed in March 2020)

SveMed+

1     palliative       1177

2     palliative care 1175
 3     terminal care   1481
 4     hospice care    202
 5     terminal        1219
 6     terminally ill  269
 7     terminally      1219
 9     palliative      1180
 10    hospice 208
 11    #1 OR #2 OR #3 OR #4 OR #5 OR #6 OR #7 OR #9 OR #10     2202
 12    colo*   1498
 13    colorectal neoplasms    843
 14    rectal neoplasms        319
 15    colonic neoplasms       327
 16    kolon*  310
 17    rektal* 80
 18    rectal* 398
 19    #12 OR #13 OR #14 OR #15 OR #16 OR #17 OR #18   1757
 20    #11 AND #19     28
 21    #11 AND #19 Limits: granskning:"peer reviewed" 23
 22    #11 AND #19 Limits: granskning:"peer reviewed" AND year:[2009 TO>2019]  3
