# Supplementary figures and images for: Health-related quality of life in patients with colorectal cancer in the palliative phase: a systematic review and meta-analysis
Source: BMC Palliat Care. 2021 Sep 16;20:144. doi: 10.1186/s12904-021-00837-9 (PMC8447559; doi:10.1186/s12904-021-00837-9)

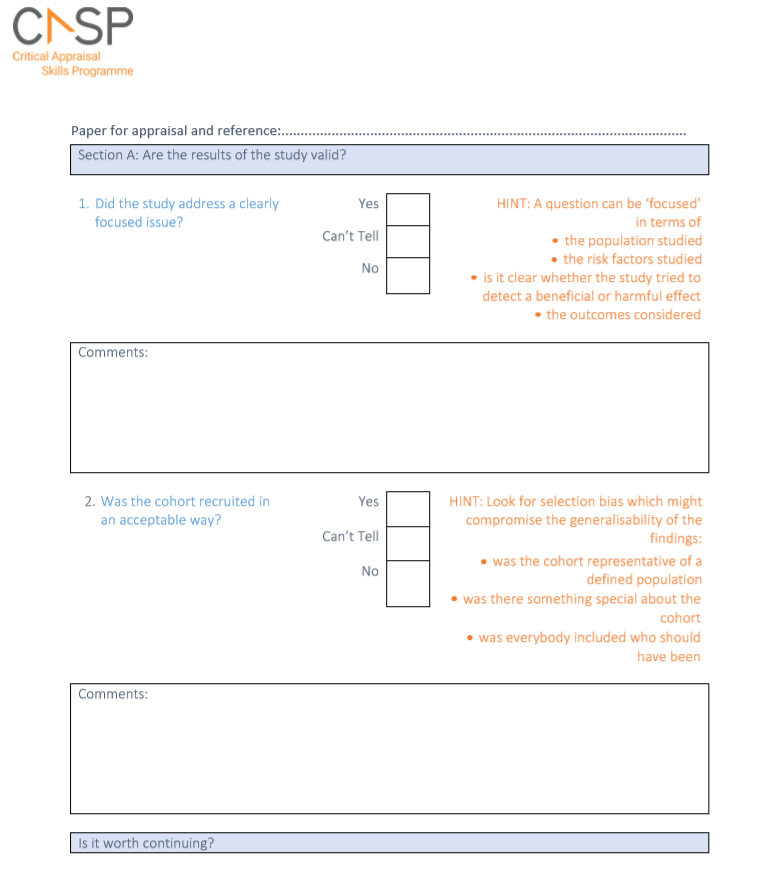
Additional file 4: Critical Appraisal Skills Programme checklist


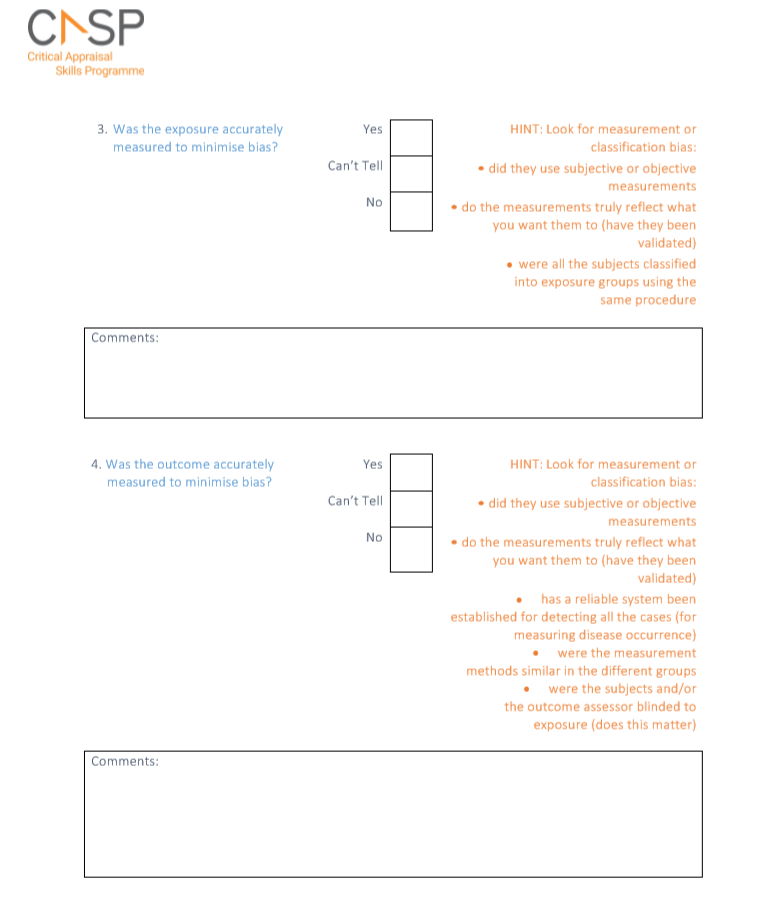


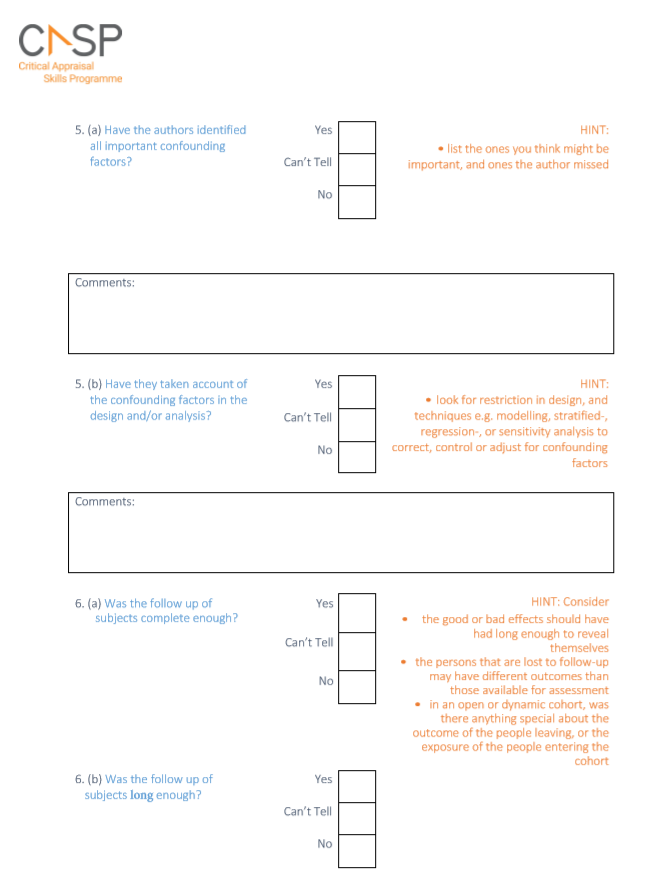


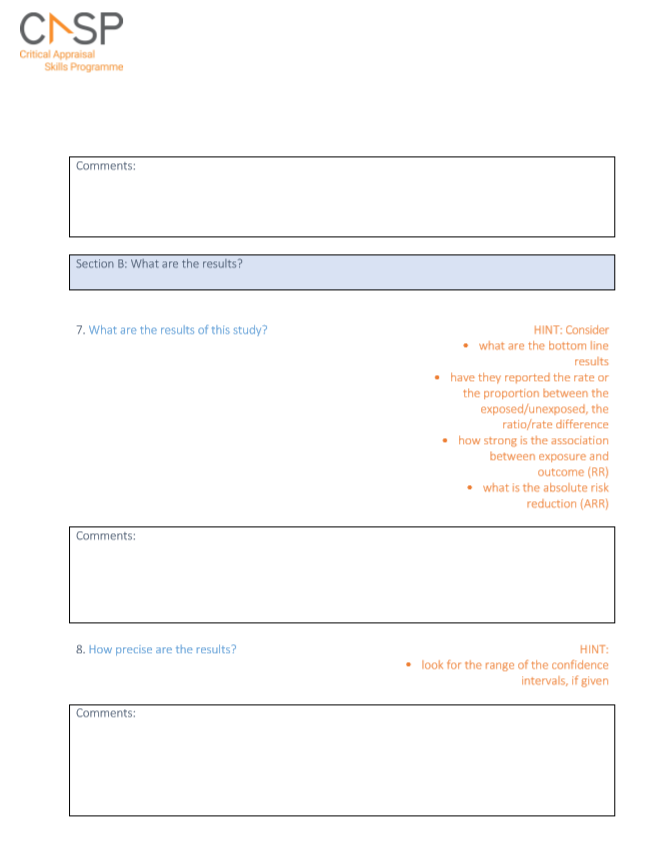


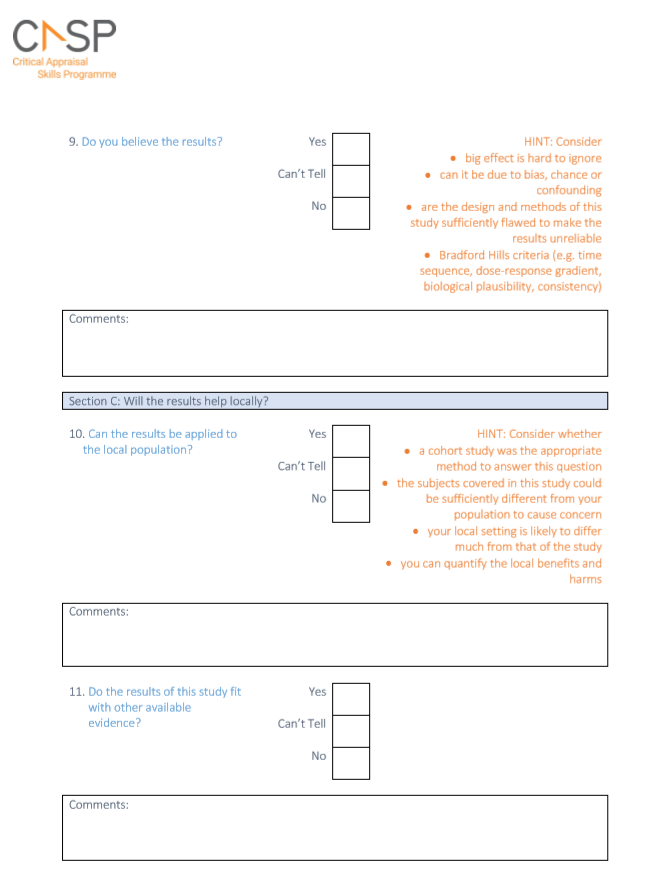


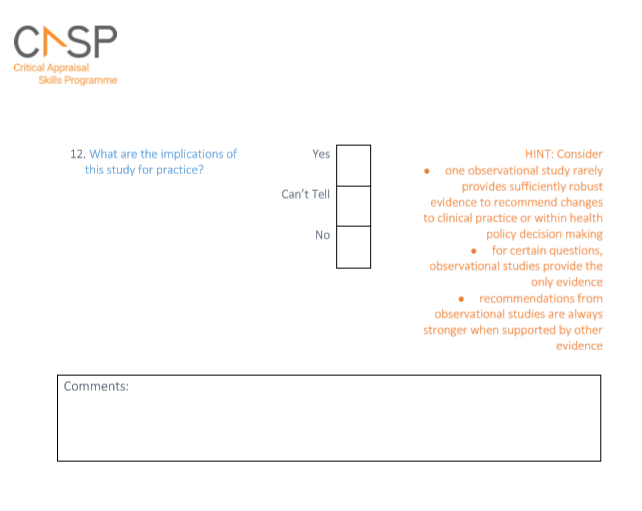

Supplement: Supplementary file 4 — Additional file 4. [file 12904_2021_837_MOESM4_ESM.docx]
