# Supplementary material for: Health-related quality of life in patients with colorectal cancer in the palliative phase: a systematic review and meta-analysis
Source: BMC Palliat Care. 2021 Sep 16;20:144. doi: 10.1186/s12904-021-00837-9 (PMC8447559; doi:10.1186/s12904-021-00837-9)
Supplement: Supplementary file 5 — Additional file 5. [file 12904_2021_837_MOESM5_ESM.docx]

**Additional file 5: Sensitivity Analysis**

**Pre-treatment:**

**Adamowicz and Baczkowska-Waliszewska (31) excluded:**

**Stein et al. (32) excluded:**

**Pre-treatment:**

**Adamowicz and Baczkowska-Waliszewska (31) excluded:**

**Pre-treatment continuing:**

**Adamowicz and Baczkowska-Waliszewska (31) excluded:**

**Adamowicz and Baczkowska-Waliszewska (31) excluded continuing:**
